# Family medicine trainees’ clinical experience of chronic disease during training: a cross-sectional analysis from the registrars’ clinical encounters in training study

**DOI:** 10.1186/s12909-014-0260-7

**Published:** 2014-12-10

**Authors:** Parker Magin, Simon Morgan, Kim Henderson, Amanda Tapley, Patrick McElduff, James Pearlman, Susan Goode, Neil Spike, Caroline Laurence, John Scott, Allison Thomson, Mieke van Driel

**Affiliations:** Discipline of General Practice, the University of Newcastle, Callaghan, 2308 NSW Australia; General Practice Training Valley to Coast, Mayfield, Australia; School of Medicine and Public Health, The University of Newcastle, Callaghan, Australia; Hunter Translational Cancer Research Unit, School of Medicine and Public Health, the University of Newcastle, Callaghan, Australia; Victorian Metropolitan Alliance, Melbourne, Australia; Discipline of General Practice, The University of Adelaide, Adelaide, Australia; Academic Discipline of General Practice, The University of Queensland, Brisbane, Australia

**Keywords:** Family practice, Education, Medical, Graduate, Chronic disease, Physician's practice patterns

## Abstract

**Background:**

A broad case-mix in family physicians’ (general practitioners’, GPs’) vocational trainee experience is deemed essential in producing competent independent practitioners. It is suggested that the patient-mix should include common and significant conditions and be similar to that of established GPs. But the content of contemporary GP trainees’ clinical experience in training is not well-documented. In particular, how well trainees’ experience reflects changing general practice demographics (with an increasing prevalence of chronic disease) is unknown. We aimed to establish levels of trainees’ clinical exposure to chronic disease in training (and associations of this exposure) and to establish content differences in chronic disease consultations (compared to other consultations), and differences in trainees’ actions arising from these consultations.

**Methods:**

A cross-sectional analysis from the Registrars’ Clinical Encounters in Training (ReCEnT) study, a cohort study of GP registrars’ (trainees’) consultations in four Australian GP training organisations. Trainees record detailed data from 60 consecutive consultations per six-month training term. Diagnoses/problems encountered are coded using the International Classification of Primary Care-2 PLUS (ICPC-2 PLUS). A classification system derived from ICPC-2 PLUS was used to define diagnoses/problems as chronic/non-chronic disease. The outcome factor for analyses was trainees’ consultations in which chronic disease was encountered. Independent variables were a range of patient, trainee, practice, consultation and educational factors.

**Results:**

Of 48,112 consultations (of 400 individual trainees), 29.5% included chronic disease problems/diagnoses. Associations of a consultation including chronic disease were the patient being older, male, and having consulted the trainee previously, and the practice routinely bulk-billing (not personally charging) patients. Consultations involving a chronic disease lasted longer, dealt with more problems/diagnoses, and were more likely to result in specialist referrals and trainees generating a personal learning goal. They were associated with less pathology tests being ordered.

**Conclusions:**

Trainees saw chronic disease less frequently than have established GPs in comparable studies. The longer duration and more frequent generation of learning goals in chronic disease-containing consultations suggest trainees may find these consultations particularly challenging. Our findings may inform the design of measures aimed at increasing the chronic disease component of trainees’ patient-mix.

## Background

The patient-mix of the clinical experiences of vocational trainees (registrars) in general practice (family medicine) is accepted to be of major educational importance in producing competent practitioners equipped to enter independent practice. It is expected that the patient-mix should include both “common and significant conditions” [1] and be similar to that of established independent general practitioners (GPs) [2]. In Australia, the subject content of Fellowship examinations of the Royal Australian College of General Practitioners is based on disease prevalence of established GPs’ consultations [3]. But the content of contemporary GP trainees’ clinical experience in training is not well-documented.

In the 1970s and 1980s a number of papers reported on the content of GP trainees’ experience, mainly in the UK [4-12]. This research was of limited scope, often being the experiences of single trainees [7-9,11] and/or single practices, [5,7-11] and lacking multivariate analysis [5,6,9-12] (or any statistical analysis [4,7,8]). But a consistent finding was an apparent deficit in trainees’ seeing patients with chronic disease (when compared with their trainers) [4-10,12]. This was also found in Dutch [13] and British [14] studies in the early 1990s.

Chronic disease is now the main health-related problem facing the world’s governments and health care systems [15]. Trends show an increased prevalence of chronic disease in general practice [16-18] during the 20 to 40 years since most of the evidence on trainees’ experience was obtained. Furthermore, the complexity of chronic disease management has increased, given the increasing prevalence of chronic disease multi-morbidity [17,18].

Contemporary research on the patient-mix of trainees is very limited, but a study from the Netherlands suggests that trainees’ exposure to chronic disease cases is greater for more senior trainees [19,20] and that trainees see less chronic disease presentations than their trainers [20]. But other predictors or associations of trainees’ chronic disease exposure, and ways in which trainees consultations involving chronic disease differ from other consultations, have not been explored either in contemporary or in older studies.

In this study we sought to establish the level of chronic disease clinical experience of a contemporary cohort of Australian general practice trainees and the associations of this chronic disease experience. We also sought to establish differences between consultations involving chronic disease presentations and those not involving a chronic disease element – both in the content of the consultation and in the actions arising from the consultation.

## Methods

This study took place within the Registrar Clinical Encounters in Training (ReCEnT) study.

### ReCEnT

ReCEnT is an ongoing multi-site cohort study of GP trainees. Participants are GP trainees training with four GP Regional Training Providers (RTPs) across four Australian states.

The methodology has been described in detail elsewhere [21]. Briefly, GP trainees undertake data collection once per six-month training term (or per twelve-month term for part-time trainees) as part of their educational program. This results in trainees collecting data on three or four occasions during their training. The data are used to provide detailed written feedback to trainees and they are encouraged to use this feedback to reflect on their clinical practice and educational and training needs. Informed consent is obtained for trainees’ de-identified data to be also used for research purposes as part of the ReCEnT study.

Initial data collection involves demographic, education, work experience, and attitudinal data from participating trainees as well as characteristics of the practice in which they are working. These parameters are recorded by each trainee, each training term.

Trainees then record the details of 60 consecutive clinical consultations per term on a paper-based encounter form. Data collection is performed mid-way through the trainee’s training term. As data collection is designed to reflect a ‘normal’ week of general practice, consultations in a specialised clinic, e.g. vaccination clinic or Pap smear clinic, are excluded. Only office-based (not home visits or nursing home visits) consultations are recorded.

The collected data encompasses four broad areas: patient demographics, diagnoses (or problems managed), investigations/management (including referral and follow-up), and educational training aspects (whether the trainee sought in-consultation advice from their trainer or information from other sources, or generated learning goals). Problems managed/diagnoses are coded according to the International Classification of Primary Care, second edition classification system (ICPC-2 PLUS) [22].

### Outcome factor

The outcome factor in this study was consultations in which a chronic disease was recorded as a diagnosis/problem by the trainee. Chronic diseases were coded via an existing classification system derived from ICPC-2 PLUS. This classification was ‘designed to identify chronic conditions managed in Australian general practice’ (the setting for our study) and is based on considerations of disease duration, prognosis, pattern, and sequelae and includes 129 complete ICPC-2 ‘rubrics’ and ICPC-2 PLUS codes from a further 20 ICPC-2 rubrics [23].

### Independent variables

Independent variables related to trainee, patient, practice and consultation.

Trainee factors were age, gender, training term, training pathway enrolled in (general or rural: rural pathway trainees train exclusively in rural locations), place of medical qualification (Australia/international), and full-time/part-time status.

Patient factors were age, gender, Indigenous (Aboriginal or Torres Strait Islander) status, new patient to the practice, and new patient to the trainee.

Practice factors included rurality/urbanicity, practice size (number of GPs), and if the practice routinely bulk-bills (that is, there is no financial cost to the patient for the consultation). Practice postcode was used to define the Australian Standard Geographical Classification-Remoteness Area (ASGC-RA) classification [24] (the degree of rurality) of the practice location and to define the practice location’s Socioeconomic Index for Area (SEIFA) Relative Index of Disadvantage [25].

Consultation factors were duration of consultation, whether a practice nurse was involved in the consultation, the number of diagnoses/problems dealt with, and if pathology was ordered or a specialist referral made. Further educational consultation factors were if the trainee sought clinical assistance during the consultation (from their supervisor/trainer, from a specialist, or from electronic or hard-copy resources) and if the trainee generated personal learning goals in the consultation.

### Statistical analysis

This was a cross-sectional analysis of patient consultations from the longitudinal ReCEnT study. Analysis was performed on the first six rounds of data collected from 2010–2012.

Percentage of trainees’ consultations involving a chronic disease was calculated, with 95% confidence intervals.

To test associations of a consultation involving chronic disease, simple and multiple logistic regression were used within a generalised estimating equations (GEE) framework to account for clustering of patients within trainees. All variables with a p value less than .20 in the univariate analysis were included in the multiple regression model.

In order to examine our research questions, three models were built, each with ‘a chronic disease being a diagnosis/problem in the consultation’ as the dependent variable:

To examine the question of associations of a trainee’s consultation involving a chronic disease, patient, practice and trainee independent variables were entered in the regression model.

To examine the question of in which ways the content of consultations involving chronic disease differs from other consultations, the above variables were entered in a model along with the following additional variables: consultation duration, sources of clinical assistance accessed by the trainee during the consultation, whether a practice nurse was involved in the consultation, and the number of problems dealt with in the consultation.

To examine the question of whether actions arising from consultations involving chronic disease differ from those arising from other consultations, all variables entered in the previous two models were entered in a new model along with the following additional variables: learning goals generated by the trainee, specialist referrals made and number of pathology tests ordered.

The rationale for the building of the three models was that whether a patient presents for a consultation with a chronic disease will plausibly be influenced by patient, trainee and practice factors, but evaluation of these influences may be compromised by inclusion in the model of factors operating once the consultation is progressing. Similarly, evaluation of the content of the consultation may be compromised by the inclusion in this model of actions arising from the consultation.

**Figure 1:**
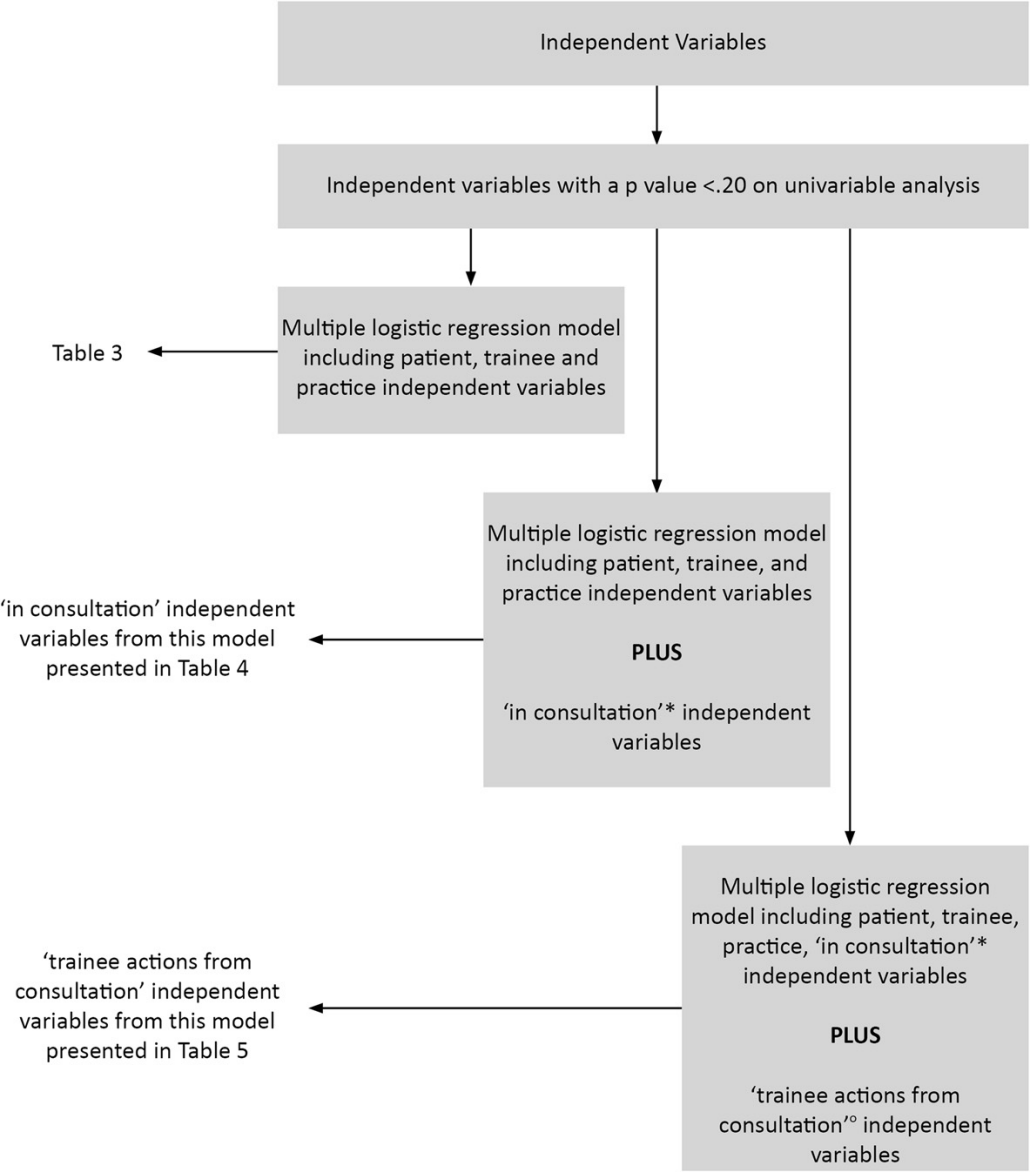
**Analysis flow-chart for outcome factor-“Chronic Disease Diagnosis/Problem”.**

The overall approach to the regression analyses is presented in Figure 1.

Statistical analyses used SAS v9.3. Predictors were considered statistically significant if the p-value < 0.05

### Ethics approval

The ReCEnT project has approval from the University of Newcastle Human Research Ethics Committee, Reference H-2009-0323.

## Results

400 individual trainees (response rate 94.7%) contributed 831 trainee-rounds of data (including details of 48,112 individual consultations).

The demographics of the participating trainees and practices are presented in Table 1.

**Table 1:** **Participating registrar (trainee), registrar-term and practice characteristics**

**Variable**	**Class**	**n % (95% CIs) or Mean (SD)**
Registrar variables (n = 400)		
Registrar gender	Male	125 31.3% (26.7-35.8)
	Female	275 68.8% (64.2-73.3)
Pathway registrar enrolled in	General	310 77.7% (73.6-81.8)
	Rural	89 22.3% (18.2-26.4)
Qualified as a doctor in Australia	No	106 26.8% (22.4-31.1)
	Yes	290 73.2% (68.9-77.6)
Registrar works fulltime	No	175 21.4% (18.6-24.2)
	Yes	642 78.6% (75.8-81.4)
Registrar age (years)	Mean (SD)	33.03 (6.73)
Registrar-term or practice-term variables (n = 831)		
Registrar training term	Term 1	333 40.1% (36.7-43.4)
	Term 2	290 34.9% (31.7-38.1)
	Term 3	156 18.8% (16.1-21.4)
	Term 4	52 6.3% (4.6-7.9)
Registrar worked at the practice previously	No	541 71.2% (68.0-74.4)
	Yes	219 28.8% (25.6-32.0)
Does the practice routinely bulk bill	No	691 84.7% (82.2-87.2)
	Yes	125 15.3% (12.8-17.8)
Number of GPs working at the practice	1-4	260 31.8% (28.6-35.0)
	5-9	445 54.5% (51.0-57.9)
	Ten or more	112 12.7% (11.3-16.1)
Rurality of practice	Major City	454 54.8% (51.4-58.2)
	Inner regional	276 33.3% (30.1-36.5)
	Outer regional or remote	99 11.9% (9.7-14.2)
SEIFA* Index (decile) of practice	Mean (SD)	989.2 (68.5)

*Socioeconomic Index for Area (SEIFA) Relative Index of Disadvantage.

Of trainees’ consultations, 29.5% (95% CI 29.1, 29.9), included a chronic disease diagnosis or problem.

The most common chronic diseases encountered were uncomplicated hypertension (5.7% of all consultations), depressive disorder (4.2%), lipid disorder (2.5%), asthma (2.2%), and oesophageal disease (1.7%). The associations of a consultation including a chronic disease are presented in Table 2.

**Table 2:** **Characteristics associated with the consultation including a chronic disease**

		**Chronic disease**		
**Variable**	**Class**	**No (n = 33921)**	**Yes (n = 14191)**	**P-value**
Registrar’s (trainees’) training term	Term1	13590 (71%)	5630 (29%)	0.12
	Term 2	11987 (72%)	4690 (28%)	
	Term 3	6281 (69%)	2833 (31%)	
	Term 4	2063 (67%)	1038 (33%)	
Registrar gender	Male	11171 (71%)	4584 (29%)	0.54
	Female	22750 (70%)	9607 (30%)	
Pathway registrar enrolled in	General	26199 (71%)	10945 (29%)	0.51
	Rural	7609 (71%)	3180 (29%)	
Qualified as a doctor in Australia	No	9488 (71%)	3805 (29%)	0.63
	Yes	24096 (70%)	10264 (30%)	
Registrar works fulltime	No	7013 (70%)	2996 (30%)	0.30
	Yes	26378 (71%)	11004 (29%)	
Registrar age (years)	mean (SD)	32.9 (6.9)	32.8 (6.7)	0.82
Registrar year of graduation	mean (SD)	2004 (6)	2004 (6)	0.43
Registrar worked at the practice previously	No	22514 (72%)	8810 (28%)	0.049
	Yes	8660 (68%)	4061 (32%)	
Does the practice routinely bulk bill	No	28318 (71%)	11737 (29%)	<0.001
	Yes	5000 (69%)	2271 (31%)	
Number of GPs working at the practice	1-4	10182 (67%)	4940 (33%)	<0.001
	5-9	18394 (71%)	7370 (29%)	
	Ten or more	4777 (74%)	1705 (26%)	
Rurality of practice	Major city	19023 (72%)	7305 (28%)	<0.001
	Inner regional	10966 (69%)	4965 (31%)	
	Outer regional or Remote	3846 (67%)	1898 (33%)	
SEIFA Index (decile) of practice	mean (SD)	6.11 (2.48)	5.82 (2.44)	<0.001
Patient age (years)	<20	9552 (87%)	1374 (13%)	<0.001
	20 to <40	9839 (76%)	3093 (24%)	
	40 to <60	7916 (63%)	4609 (37%)	
	60 to <80	4798 (56%)	3827 (44%)	
	80+	1354 (56%)	1070 (44%)	
Patient gender	Male	12746 (70%)	5416 (30%)	0.088
	Female	20524 (71%)	8479 (29%)	
Patient aboriginal or torres strait islander	No	33619 (71%)	14034 (29%)	0.96
	Yes	302 (66%)	157 (34%)	
New patient to the registrar and/or practice	Seen registrar before	13167 (64%)	7464 (36%)	<0.001
	New patient to registrar	18110 (75%)	5949 (25%)	
	New patient to practice	2644 (77%)	778 (23%)	
Patient by practice nurse seen during consultation	No	33211 (70%)	13898 (30%)	0.073
	Yes	710 (71%)	293 (29%)	
Any referral made during consultation	No	29340 (73%)	10839 (27%)	<0.001
	Yes	4581 (58%)	3352 (42%)	
Registrar generated learning goals	No	27286 (72%)	10381 (28%)	<0.001
	Yes	5919 (62%)	3559 (38%)	
Registrar sought assistance from any source during consultation	No	28337 (71%)	11487 (29%)	<0.001
	Yes	5584 (67%)	2704 (33%)	
Duration of consultation (hours)	mean (SD)	0.26 (0.14)	0.32 (0.17)	<0.001
Number of problems addressed during consultation	mean (SD)	1.37 (0.66)	2.00 (0.95)	<0.001
Number of pathology tests ordered	mean (SD)	0.69 (1.87)	1.09 (2.28)	<0.001
Number of imaging tests ordered	mean (SD)	0.13 (0.39)	0.16 (0.44)	<0.001

The regression model with dependent variable of ‘a consultation including a chronic disease’, and including patient, trainee and practice independent variables, is presented in Table 3. The only trainee factor significantly associated with the consultation including a chronic disease was the trainee’s training term, with consultations of Term 2 trainees being less likely than those of Term 1 trainees to include a chronic disease (OR 0.86). The only practice factor significantly associated with trainees seeing more chronic disease was the practice routinely bulk-billing patients (the patient doesn’t incur a personal cost for the consultation, OR 1.12). There was a non-significant trend (p = .056) for trainees working in practices in areas of greater socioeconomic disadvantage to see more chronic disease (OR 0.98 for each decile of the SEIFA index). Patient factors significantly associated with the consultation including chronic disease were greater age (OR 4.75 for the oldest versus youngest age-band) and male gender (female versus male OR 0.90). A patient having been seen by the trainee previously and the patient not being new to the practice were both associated with the consultation having a chronic disease element (ORs of 0.68 and 0.70 for new patient to the practice and new patient to the trainee, respectively).

**Table 3:** **Characteristics associated with the consultation including a chronic disease: model including registrar (trainee), patient and practice variables**

		**Univariate**		**Adjusted**	
**Variable**	**Class**	**OR (95% CI)**	**P**	**OR (95% CI)**	**P**
Registrar’s training term	Term 2	0.93 (0.87, 0.99)	0.031	0.86 (0.80, 0.93)	<0.001
Referent: Term 1	Term 3	1.00 (0.91, 1.10)	0.98	0.98 (0.89, 1.08)	0.65
	Term 4	0.96 (0.82, 1.11)	0.56	0.87 (0.73, 1.04)	0.12
New patient to the registrar and/or practice	New patient to practice	0.63 (0.60, 0.66)	<0.001	0.68 (0.65, 0.71)	<0.001
Referent: Seen by registrar before	New patient to registrar	0.56 (0.51, 0.62)	<0.001	0.70 (0.62, 0.78)	<0.001
Registrar worked at the practice previously	Yes	1.09 (1.00, 1.18)	0.050	1.08 (0.99, 1.18)	0.091
Does the practice routinely bulk bill	Yes	1.21 (1.09, 1.34)	<0.001	1.12 (1.00, 1.26)	0.047
Rurality of practice	Inner regional	1.17 (1.07, 1.27)	<0.001	1.08 (0.98, 1.18)	0.12
Referent: Major city	Outer regional/Remote	1.23 (1.07, 1.41)	0.0037	1.07 (0.93, 1.24)	0.33
Number of GPs working at the practice/post	5-9	0.85 (0.78, 0.92)	<0.001	0.92 (0.85, 1.01)	0.081
Referent: 1-4	Ten or more	0.78 (0.68, 0.90)	<0.001	0.87 (0.75, 1.01)	0.063
SEIFA Index (decile) of practice		0.96 (0.95, 0.98)	<0.001	0.98 (0.97, 1.00)	0.056
Patients age (years)	20 to <40	2.17 (2.01, 2.34)	<0.001	2.10 (1.94, 2.29)	<0.001
Referent: < 20	40 to <60	3.89 (3.61, 4.20)	<0.001	3.75 (3.45, 4.06)	<0.001
	60 to <80	5.14 (4.72, 5.59)	<0.001	4.82 (4.40, 5.28)	<0.001
	80+	5.03 (4.48, 5.65)	<0.001	4.75 (4.18, 5.38)	<0.001
Patient gender	Female	0.96 (0.92, 1.01)	0.088	0.90 (0.86, 0.94)	<0.001

The significant ‘consultation’ associations of having a chronic disease addressed in the consultation (adjusted for patient, trainee and practice) are presented in Table 4. Consultations involving chronic disease were associated with longer duration (OR 4.17 for each extra hour) and more problems/diagnoses being addressed (OR 2.15 for each extra problem addressed).

**Table 4:** **Characteristics associated with the consultation including a chronic disease: consultation variables (in a model adjusted for registrar (trainee), patient, and practice variables)**

		**Univariate**		**Adjusted**	
**Variable**	**Class**	**OR (95% CI)**	**P**	**OR (95% CI)**	**P**
Registrar sought help from any source during the consultation	Yes	1.18 (1.11, 1.26)	<0.001	1.03 (0.96, 1.11)	0.3704
Duration of consultation (hours)		14.3 (11.8, 17.4)	<0.001	4.17 (3.32, 5.25)	<0.001
Number of problems addressed during the consultation		2.50 (2.41, 2.59)	<0.001	2.15 (2.07, 2.23)	<0.001

In terms of actions arising out of consultations (see Table 5), a consultation’s involving chronic disease was significantly associated (adjusted for patient, trainee, practice and consultation variables) with the trainee generating learning goals (OR 1.36) and making referrals (OR 1.45). Consultations involving chronic disease were also associated with ordering lesser numbers of pathology tests (OR 0.99 for each extra pathology test).

**Table 5:** **Characteristics associated with the consultation including a chronic disease: ‘registrar action’ variables (in a model adjusted for registrar (trainee), patient, practice and consultation variables)**

		**Univariate**		**Adjusted**	
**Variable**	**Class**	**OR (95% CI)**	**P**	**OR (95% CI)**	**P**
Registrar generated learning goals during the consultation	Yes	1.70 (1.60, 1.81)	<0.001	1.36 (1.26, 1.46)	<0.001
Registrar made any referral during the consultation	Yes	1.87 (1.78, 1.98)	<0.001	1.45 (1.36, 1.56)	<0.001
Number of pathology tests ordered		1.08 (1.07, 1.09)	<0.001	0.99 (0.97, 1.00)	0.040

## Discussion

### Summary of main findings and comparison with existing literature

We found that 29.5% of trainee consultations included a chronic disease diagnosis or problem and that the most commonly encountered chronic diseases were hypertension, depressive disorder, lipid disorder, asthma, and oesophageal disease.

Associations of a consultation including chronic disease were the patient being older, male, and having consulted the trainee previously, and the practice routinely bulk-billing (not personally charging) patients. Consultations involving a chronic disease lasted longer, dealt with more problems/diagnoses, and were more likely to result in specialist referrals and trainees generating a personal learning goal. The effect sizes of these differences were clinically as well as statistically significant.

Our rate of trainees’ consultations including chronic disease (29.5%) cannot be directly compared with the only other contemporary finding, that of de Jong et al. (8.7% in first year trainees and 10.8% in third year trainees) [20] as the chronic disease classification systems employed in the two studies are different. It can, however, be directly compared with that of Australian established GPs (in a study with methodology similar to our study and using the same method of classification of chronic disease) which has been found to be 40.7% in male patients and 38.7% in females [26]. Thus, in Australia, trainees see chronic disease in a considerably smaller proportion of consultations than their established GP colleagues.

Our finding of an association of chronic disease exposure with the trainees being in their first term of training is in contrast to the Dutch study of de Jong et al. [19,20] which found senior trainees saw more chronic disease than junior trainees. But this may reflect the structures of the two training programs, with the curriculum in the Netherlands focussing on chronic care in the final year of training [19].

There is no literature with which to compare our findings of other associations of trainees’ chronic disease exposure.

### Strengths and limitations

The generalizability of the study is strong, given the participation of four of Australia’s 17 RTPs, in four of Australia’s six states, the trainee demographics resembling those of Australian trainees overall, [27-29] and the reach of practice location across all urban/rural classifications.

The response rate (94.7%) and statistical power provided by 48,112 consultations are also strengths of our study. The response rate is singularly high for a study recruiting GPs [30]. The large sample size and the large number of independent variables collected enables a detailed multivariate examination of the associations of trainees’ consultations with patients with chronic diseases.

A limitation of the study, however, is the fact that our dichotomous outcome factor, ‘chronic disease/not chronic disease’, is a fairly crude construct and cannot reflect the complexities of individual and very different chronic diseases, nor the complexity consequent upon multi-morbidity in many patients with chronic disease.

### Implications for educational practice

An important consideration is, ‘Does the level of trainees’ exposure to chronic disease matter?’ Authors and commentators certainly think so [20,31]. Research on undergraduate general practice placements suggests that patient mix is a factor in the ‘effectiveness’ of the placement (though not as important as supervision quality) [32] and in ‘instructional quality’ [33]. In undergraduate internal medicine placements, exposure to ‘core problems’ is associated with improved clinical performance [34]. But there is very little evidence concerning the effect of patient-mix on GP vocational trainee performance or competence. De Jong et al. have found that volume of dermatology and psychosocial consultations is associated with GP trainees’ self-assessed self-efficacy in these clinical areas [35]. But further evidence in general practice, and evidence specifically related to chronic disease in general practice, is lacking.

Despite this, there is strong opinion that chronic disease exposure commensurate with that of established GPs is desirable for trainees [1]. Some of our study’s findings are relevant here. Consultations involving chronic disease were significantly longer. This may suggest that trainees found these consultations (and, by implication, chronic disease management) challenging. As well as an increased duration of consultations, there were more problems/diagnoses dealt with in consultations including chronic disease, but the association of chronic disease with consultation duration remained strongly significant in the adjusted model (Table 4). Even though there was no increase in recourse to trainer or other sources of in-consultation assistance, chronic disease consultations were significantly more likely to prompt the trainee to generate learning goals. This also suggests that chronic diseases remain challenging for trainees. Increasing trainees’ experience dealing with this challenging situation, by increasing the chronic disease component of the training patient-mix, may thus be desirable.

The finding of greater exposure to chronic disease being associated with trainees working in practices that routinely bulk-bill (that is, there is no financial cost to the patient for the consultation) probably reflects both the increased need for care and the limited capacity of patients with chronic disease (often elderly and/or disabled by their disease) to pay for their care. The association of a consultation involving chronic disease and ordering less pathology tests was unexpected. The effect size is not large, but this may represent a lack of continuity of care - patients with chronic disease finding it convenient to see registrars for ‘one-off’ routine writing of scripts and referrals but reserving ongoing management (that would involve investigation) for a more senior GP in the practice [36,37].

How an increase in trainee exposure to chronic disease might be achieved is problematic. As early as 1980 a single-practice UK study sought to direct patients with chronic disease to the trainee rather than the trainer, but did not statistically test the outcomes [8]. Practice receptionists influence the patient-mix of trainees, [38] but a trial of receptionists’ ‘steering‘of certain patient groups to trainees didn’t demonstrate a difference in trainee patient-mix [35].

‘Directing’ patients with chronic disease to trainees may be especially problematic – there is evidence of strong preference in older patients to see their ‘usual’ GP rather than a trainee, and to have continuity of care, for chronic diseases [37]. Our study found chronic disease patients were more likely than non-chronic disease patients to have seen the trainee before. This is consistent with a desire for continuity of care, even if that care is delivered by trainees. It also suggests trainees spending more than one term in a single practice may enhance chronic disease exposure.

### Implications for future research

Previous Australian research suggests patients’ reluctance regarding chronic disease management by trainees may be attenuated by trainers maintaining oversight of trainees’ management [36]. Thus, a model for future chronic disease management in training practices might be ‘shared care’ involving trainee-delivered continuity of care with trainer oversight. Designing and implementing that model could take into account our findings of differences in trainees’ chronic disease exposure associated with practice billing policy and the longer consultation time already inherent in chronic disease-containing consultations. It should also take into account the apparent challenging nature for trainees of chronic-disease-containing consultations. Trialling such a model of care on the chronic disease content of trainees’ clinical experience is a suitable area for future research.

## Conclusions

Trainees see chronic disease in a smaller proportion of clinical consultations than that of established GPs in a comparable study. Current vocational training policies suggest these proportions should be equivalent. The longer duration and more frequent generation of learning goals in chronic disease-containing consultations in our study suggest trainees may find these consultations particularly challenging. Our findings may inform the design of measures aimed at increasing the chronic disease component of trainees’ patient-mix.

## Abbreviations

ASGC-RA: Australian standard geographical classification-remoteness area)
GEE: Generalised estimating equations
GP: General practitioner
ICPC-2: International classification of primary care –second edition
ReCEnT: Registrars’ clinical encounters in training
RTPs: Regional training providers
SEIFA: Socioeconomic index for area relative index of disadvantage
UK: United Kingdom
